# Deep saliency models learn low-, mid-, and high-level features to predict scene attention

**DOI:** 10.1038/s41598-021-97879-z

**Published:** 2021-09-16

**Authors:** Taylor R. Hayes, John M. Henderson

**Affiliations:** 1 Center for Mind and Brain, University of California, Davis, 95618 USA; 2 Department of Psychology, University of California, Davis, 95616 USA

**Keywords:** Neuroscience, Psychology

## Abstract

Deep saliency models represent the current state-of-the-art for predicting where humans look in real-world scenes. However, for deep saliency models to inform cognitive theories of attention, we need to know *how* deep saliency models prioritize different scene features to predict where people look. Here we open the black box of three prominent deep saliency models (MSI-Net, DeepGaze II, and SAM-ResNet) using an approach that models the association between attention, deep saliency model output, and low-, mid-, and high-level scene features. Specifically, we measured the association between each deep saliency model and low-level image saliency, mid-level contour symmetry and junctions, and high-level meaning by applying a mixed effects modeling approach to a large eye movement dataset. We found that all three deep saliency models were most strongly associated with high-level and low-level features, but exhibited qualitatively different feature weightings and interaction patterns. These findings suggest that prominent deep saliency models are primarily learning image features associated with high-level scene meaning and low-level image saliency and highlight the importance of moving beyond simply benchmarking performance.

## Introduction

Our everyday visual world contains too much information to take in all at once, so we filter our visual world by moving our eyes to prioritize some regions over others. But how do humans know where to look to efficiently build a representation and understanding of complex, real-world scenes? One approach to answering this question is to construct computational models that predict where people look in scenes. Deep convolutional neural network models of saliency (i.e., ‘deep saliency models’) reflect the current state-of-the-art computational models for predicting where humans look in scenes^1^. Although deep saliency models often generate very good predictions of human behavior, relatively little is known about *how* they predict where people look. For deep saliency models to inform cognitive theories of attention requires a better understanding of what deep saliency models are learning about where people look in scenes.

To begin, it is helpful to distinguish *deep saliency models* from *image saliency models*. Image saliency models are computed from the scene image alone by combining local contrasts in low-level, pre-semantic image features like color, luminance, and orientation across multiple spatial scales^2–6^. For example, a bright red flower surrounded by green grass would be a region that would be predicted by an image saliency model to capture attention. In comparison, deep saliency models use a data-driven approach that combines deep convolutional neural networks trained on large object recognition datasets (e.g., VGG-16 or VGG-19^7^) with additional network layers that are subsequently trained on human fixation data^8^. Within this approach, deep saliency models learn a mapping between the pre-trained object recognition features and the human fixation data they are trained on. Therefore, a critical difference is that image saliency models only use low-level image features to generate their predictions, whereas deep saliency models might use some combination of low-, mid-, and high-level features to generate their predictions because they are trained using both object recognition and human fixation data^9^. Therefore, in order to understand the factors that drive deep saliency models, we will need to assess the association between attention, low-, mid-, and high-level scene information, and deep saliency model output.

A large body of previous research has shown an association between pre-semantic, low-level stimulus features and attention. Early theories of attention focused on the role of low-level feature differences in capturing attention and were based on experiments using simple stimuli like lines and/or basic shapes that varied in low-level features like orientation, color, luminance, texture, shape, or motion^10–12^. These early theories were formalized into computational image ‘saliency’ models that combined the different low-level feature maps based on mechanisms observed in early visual cortex such as center-surround dynamics to generate quantitative predictions in the form of ‘saliency maps’^4,5,13–15^. Image saliency maps were shown to be significantly correlated with where people looked in scenes^2–6^. This foundational work spawned a large number of computational image saliency models (e.g., Graph-based saliency model^3^; Adaptive Whitening Saliency^16^; RARE^17^, Attention based on Information Maximization^18^) that each generate image saliency maps in different ways to improve their overall biological plausibility and/or performance on scene benchmark datasets^1^. Given the extensive theoretical, biological, and computational work on the role of low-level features in guiding attention, it will be important to quantify the degree to which low-level features are associated with deep saliency model performance.

Mid-level vision is thought to play a role in organizing low-level features in specific ways (e.g., Gestalt principles) that facilitate higher-level recognition processes^19–23^. However, there has been very little work on the role that mid-level features play in guiding overt attention in scenes^9^. A recent study^9^ showed that two different proposed mid-level features, local symmetry and contour junctions, contributed to category-specific scene attention during a scene memorization task in grayscale scenes and line drawings. The mid-level scene category predictions were also computed over discrete temporal time bins, and the results suggested that symmetry contributed to both early bottom-up and later top-down guidance, while junctions contributed mostly to later top-down guidance^9^. Therefore, in the present work, it will also be useful to directly quantify the association between attention, mid-level features (i.e., local symmetry and contour junctions), and deep saliency model output.

Finally, there is a growing literature suggesting that high-level semantic density plays an important role in guiding attention in real-world scenes^24–30^. Much of this work has shown that high-level semantics often overrides low-level salience^25–28,31^. While many semantic scene studies manipulate a single or small number of objects in each scene, it is also possible to use human raters to rate the meaningfulness of scene regions based on how informative or recognizable regions are to generate a continuous distribution of local semantic density across the entire scene (i.e., a meaning map^26^). Meaning maps have been shown to be one of the strongest predictors of where people look in scenes across a wide variety of scene viewing tasks including scene memorization^26,27^, visual search^32^, free viewing^33^, scene description^34^, and saliency search^35^. Therefore, in the present study it will be important to assess the degree to which the image features learned by deep saliency models are associated with high-level meaning.

In the present work, we had two main goals. First, we sought to replicate and extend recent results demonstrating that prominent deep saliency models (MSI-Net^36^; DeepGaze II^37^; and SAM-ResNet^38^) provide excellent predictions of human attention during free-viewing of scenes. We addressed this goal using a large eye movement dataset in which 100 participants viewed 100 scenes and performed active scene viewing tasks rather than passive free-viewing. Our analyses explicitly accounted for center bias^39,40^ and the random effects of viewer and scene using a mixed effects modeling approach^30,41^. Second, and more importantly, we determined which features prominent deep saliency models prioritize to predict attention by modeling the association between deep saliency model output and attention to low-level saliency^3,42^, mid-level symmetry and junctions^43,44^, and high-level scene meaning^26^. Without an understanding of how deep saliency models prioritize different scene features to generate their predictions, we have no way to determine how human-like deep saliency models actually are. Therefore, the present work seeks to build a bridge between deep saliency models and human eye movement behavior beyond just overall prediction.

## Results

**Figure 1:**
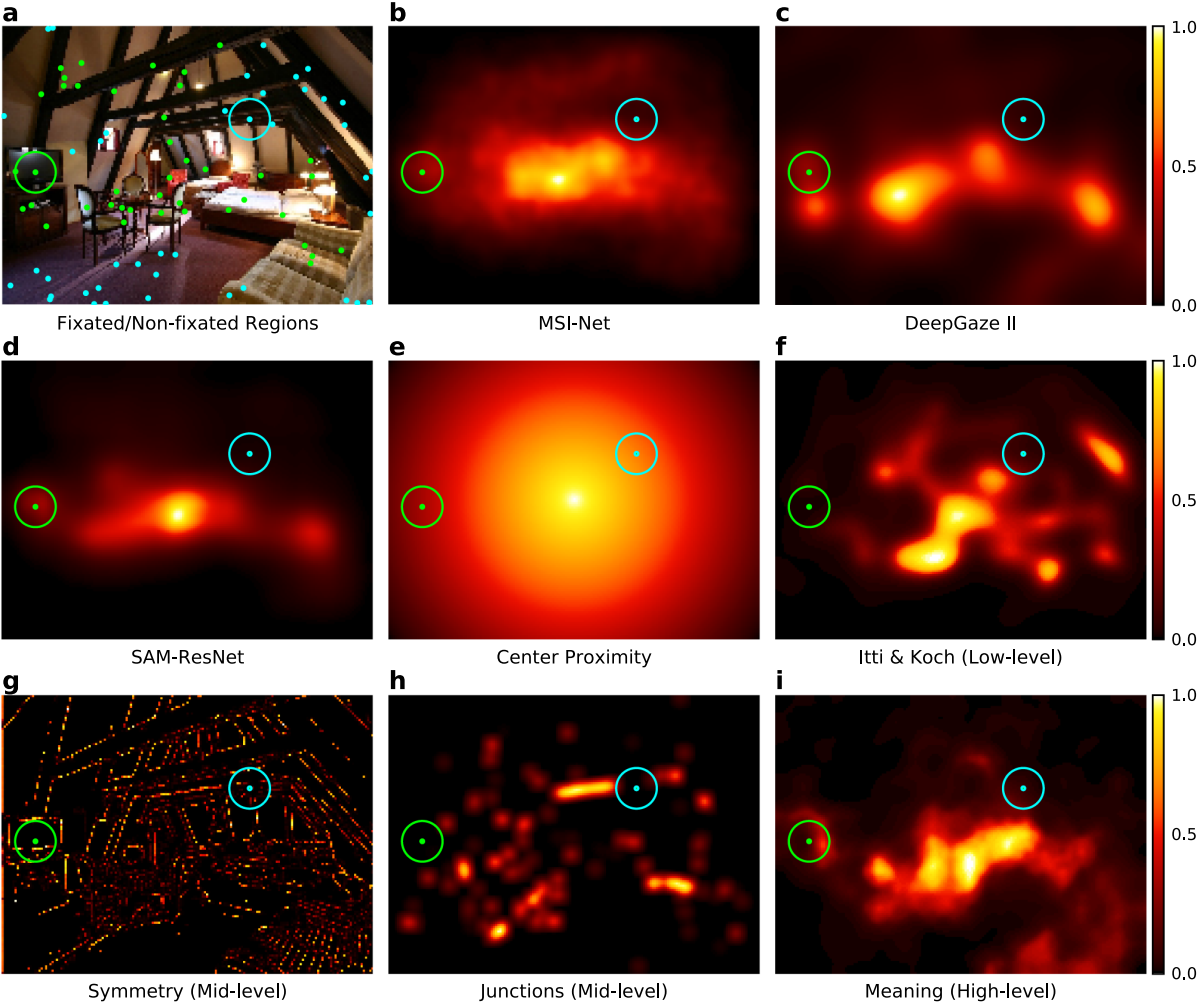
Scene with the fixated and non-fixated regions for a typical subject, and the corresponding deep saliency and feature maps. (**a**) The solid green dots show the scene locations where the subject fixated, and the solid cyan dots indicate randomly sampled non-fixated regions that represent where this subject did not look in this scene. Together these locations provide an account of the regions in this scene that did and did not capture this subject’s attention. Each fixated and non-fixated location was then used to compute a mean value for each deep saliency model map (**b**–**d**) and feature map (**e**–**i**) across a $3^{\circ }$ window (shown as circles around one example fixated and non-fixated location in each map). All the heat maps were scaled (0–1) and plotted in Matplotlib^45^ (3.4.2, https://matplotlib.org/) from their respective source maps (see “Methods” for details).

We first measured the strength of the association between where subjects looked in each scene and each respective deep saliency model. We did this by examining whether fixations on a scene region could be accounted for by the mean deep saliency and center proximity values (averaged across a $3^{\circ }$ window) at fixated and non-fixated locations. We examined each deep saliency model separately by fitting a separate logistic general linear mixed effects (GLME) model for each deep saliency model. Within each GLME model, whether a region was fixated (1) or not (0) was the dependent variable and the scene region’s mean deep saliency model value (MSI-Net Fig. 1b; DeepGaze2, Fig. 1c; SAM-ResNet Fig. 1d), mean center proximity value (Fig. 1e), and the deep saliency by center proximity interaction were treated as predictors. Subject and scene were treated as random intercepts in each GLME model. These three GLME models reflect whether fixations could be predicted by each deep saliency model while controlling for center bias and the random effects of subject and scene.

**Figure 2:**
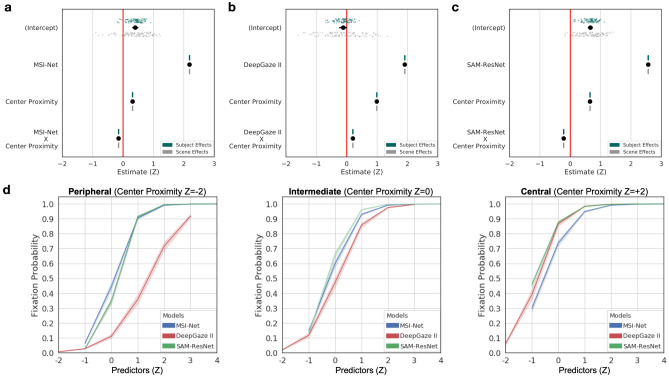
Deep saliency model general linear mixed effects model results. Whether a scene region was fixated or not was modeled as a function of the mean deep saliency model value, mean center proximity value, and their interaction as fixed effects (**a** MSI-Net; **b** DeepGaze II; **c** SAM-ResNet). The black dots with lines show the fixed effect estimates and their 95% confidence intervals. Subject (green dots) and scene (grey dots) were both accounted for in the model as random intercepts. (**d**) A line plot of the interaction between center proximity (panels) and each deep saliency model (colored lines) as a function of fixation probability. All error bands reflect 95% confidence intervals.

The results are shown in Fig. 2 and Table 1. In each GLME model, there was a significant positive fixed effect of a scene region’s deep saliency model value (MSI-Net $\beta ={2.19}$, CI [2.17, 2.20], $p<.001$; DeepGaze II, $\beta ={1.82}$, CI [1.81, 1.83], $p<.001$; SAM-ResNet, $\beta ={2.57}$, CI [2.55, 2.59], $p<.001$). Additionally, each deep saliency model interacted with center proximity (MSI-Net, $\beta ={-0.15}$, CI $[-0.16, -0.13]$, $p<.001$; DeepGaze II, $\beta ={0.16}$, CI [0.15, 0.17], $p<.001$; SAM-ResNet, $\beta ={-0.22}$, CI $[-0.24, -0.20]$, $p<.001$). These interactions are shown as a function of fixation probability in Fig. 2d). Finally, we computed the classification rates of each GLME model (MSI-Net = 0.82, DeepGaze II = 0.83, SAM-ResNet = 0.81; chance-level = 0.50), indicating that the models produced similar prediction of whether a scene region was fixated (1) or not (0). Taken together these results extend previous findings using free-viewing tasks^36–38^ and establish that MSI-Net, DeepGaze II, and SAM-ResNet also predict scene attention well in active viewing tasks (i.e., scene memorization and aesthetic judgment).

**Table 1:** Logistic general linear mixed effects model results for each deep saliency model: MSI-Net, DeepGaze II, and SAM-ResNet.

Predictors	Fixed effects					Random effects, *SD*	
	$\beta$	95% CI	*SE*	*z*-statistic	*p*	Subject	Scene
**MSI-Net**							
Intercept	0.40	[0.31 0.50]	0.05	8.05	< 0.001***	0.23	0.45
MSI-Net	2.19	[2.17 2.20]	0.01	295.36	< 0.001***	–	–
Center proximity	0.31	[0.30 0.32]	0.01	57.93	< 0.001***	–	–
MSI-Net: center proximity	− 0.15	[− 0.16 − 0.14]	0.01	− 22.65	< 0.001***	–	–
**DeepGaze II**							
Intercept	− 0.11	[-0.25 0.02]	0.07	− 1.69	0.09	0.24	0.63
DeepGaze II	1.91	[1.90 1.92]	0.01	339.92	< 0.001***	–	–
Center proximity	0.99	[0.98 1.00]	0.01	225.03	< 0.001***	–	–
DeepGaze II :center proximity	0.20	[0.19 0.21]	0.01	33.85	< 0.001***	–	–
**SAM-ResNet**							
Intercept	0.66	[0.58 0.75]	0.04	15.54	< 0.001***	0.21	0.37
SAM-ResNet	2.57	[2.55 2.59]	0.01	242.82	< 0.001***	–	–
Center proximity	0.65	[0.64 0.66]	0.01	104.63	< 0.001***	–	–
SAM-ResNet: center proximity	− 0.22	[− 0.24 − 0.20]	0.01	− 19.99	< 0.001***	–	–

Beta estimates ($\beta$), 95% confidence intervals (CI), standard errors (SE), $z-$statistic, and *p*-values (*p*) for each fixed effect and standard deviations (*SD*) for the random effects of subject and scene.

However, demonstrating that deep saliency models are strongly associated with where people look in scenes during active viewing, does not tell us anything about *how* these models predict where people look in scenes. Therefore, to gain some insight into how each deep saliency model is prioritizing different types of scene features, we turned the analysis on its head and modeled the associations between the deep saliency model values and low-, mid-, and high-level feature values for each fixated scene region. Specifically, we fit a linear mixed effects model (LME) for each deep saliency model, where the fixated mean deep saliency model values (Fig. 1b–d) were the dependent variable and the corresponding low-level (image saliency), mid-level (symmetry and junctions), high-level (meaning), and center proximity map values were treated as fixed effects (Fig. 1e–i, respectively). We also included interaction terms for center proximity with each feature type (i.e., low-, mid-, and high-level) and a term to account for the known interaction between low- and high-level scene features^26,32^. Subject and scene were treated as random intercepts in each LME model. Using this LME approach to analyze our data allowed us to measure the association between attention, each deep saliency model, and each of our defined feature maps while controlling for center bias and the random effects of subjects and scenes. Since all model terms were standarized prior to fitting each LME model, the feature-levels can be directly compared using the 95% confidence intervals within each deep saliency LME model. That is, if the 95% confidence intervals of the parameter estimate of two different fixed effects (e.g., meaning and IttiKoch saliency) do not overlap, then they are significantly different. Therefore, this approach allowed us to address our main question of interest, what do deep saliency models learn about where we look in scenes and how do they weight different types of scene features?

**Figure 3:**
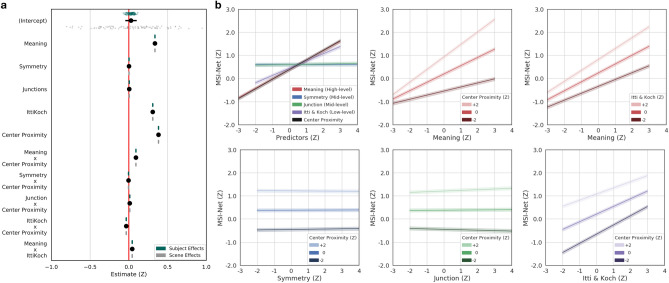
MSI-Net linear mixed effects model, marginal effects, and interactions. (**a**) Fixated MSI-Net values as a function of low-, mid-, and high-level features and interactions. The black dots with lines show the fixed effect estimates and their 95% confidence intervals. Subject (green dots) and scene (grey dots) were both accounted for in the model as random intercepts. (**b**) Line plots of all model marginal effects and all model interactions. All error bands reflect 95% confidence intervals.

**Figure 4:**
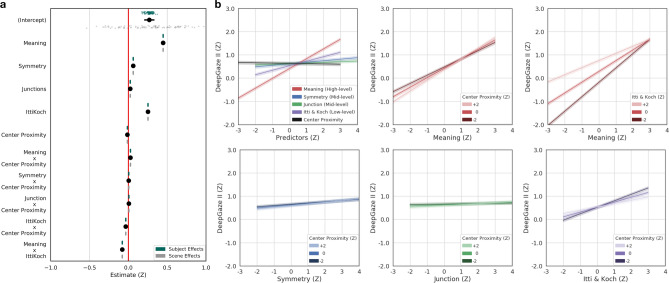
DeepGaze II LME model, marginal effects, and interactions. (**a**) Fixated DeepGaze II values as a function of low-, mid-, and high-level features and interactions. The black dots with lines show the fixed effect estimates and their 95% confidence intervals. Subject (green dots) and scene (grey dots) were both accounted for in the model as random intercepts. (**b**) Line plots of all model marginal effects and all model interactions. All error bands reflect 95% confidence intervals.

**Figure 5:**
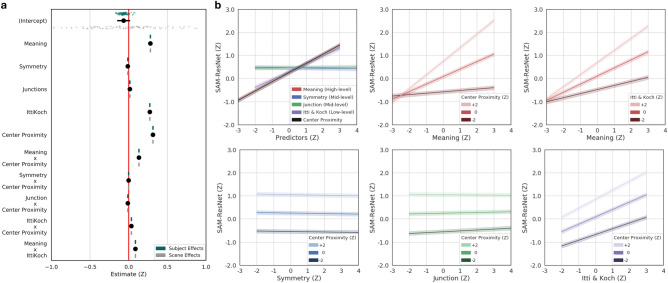
SAM-ResNet LME model, marginal effects, and interactions. (**a**) Fixated SAM-ResNet values as a function of low-, mid-, and high-level features and interactions. The black dots with lines show the fixed effect estimates and their 95% confidence intervals. Subject (green dots) and scene (grey dots) were both accounted for in the model as random intercepts. (**b**) Line plots of all model marginal effects and all model interactions. All error bands reflect 95% confidence intervals.

The feature association LME model results are shown below for MSI-Net (Fig. 3; Table 2), DeepGaze II (Fig. 4; Table 3) and SAM-ResNet (Fig. 5; Table 4). High-level meaning was the strongest predictor in MSI-Net ($\beta ={0.33}$, CI [0.33, 0.34], $p<.001$) and DeepGaze II (DeepGaze II, $\beta ={0.44}$, CI [0.44, 0.45]) followed by low-level saliency (MSI-Net: $\beta ={0.308}$, CI [0.305, 0.312], $p<.001$; DeepGaze II: $\beta ={0.253}$, CI [0.250, 0.255], $p<.001$). In SAM-ResNet, high-level meaning ($\beta ={0.28}$, CI [0.27, 0.29], $p<.001$) and low-level saliency ($\beta ={0.27}$, CI [0.27, 0.28], $p<.001$) were equally strong predictors. In all three deep saliency models, the mid-level junctions (MSI-Net $\beta ={0.006}$, CI [0.003, 0.008], $p<.001$; DeepGaze II, $\beta ={0.03}$, CI [0.02, 0.03], $p<.001$; SAM-ResNet, $\beta ={0.016}$, CI [0.012, 0.019], $p<.001$) and symmetry (DeepGaze II, $\beta ={0.06}$, CI [0.05, 0.07], $p<.01$; SAM-ResNet, $\beta ={-0.01}$, CI $[-0.02, 0.00]$, $p<.01$) features were only weakly associated with the deep saliency model values. Together these findings suggest that deep saliency models are primarily learning features associated with high-level meaning and low-level saliency, while mid-level symmetry and junctions play a more marginal role.

Based on previous work^26,27,32^ that showed a relationship between high-level meaning and low-level saliency, we included an interaction term (high-level meaning X low-level image saliency) in each of our decomposed deep saliency model analyses. The high-level by low-level interaction was significant in all three deep saliency models (MSI-Net $\beta ={0.04}$, CI [0.04, 0.05], $p<.001$; DeepGaze II, $\beta ={-0.08}$, CI $[-0.08, -0.07]$, $p<.001$; SAM-ResNet, $\beta ={0.09}$, CI [0.08, 0.09], $p<.001$), but displayed different qualitative interaction patterns. MSI-Net (Fig. 3b, top-right) and SAM-ResNet (Fig. 5b, top-right) displayed a similar interaction pattern; as a fixated region’s meaning value increased the predicted MSI-Net and SAM-ResNet values increased more quickly with greater low-level saliency. DeepGaze II displayed the opposite interaction pattern (Fig. 4b, top-right); as a fixated region’s meaning value increased the predicted DeepGaze II values increasingly were unaffected by low-level saliency. These divergent interaction patterns suggest that MSI-Net and SAM-ResNet predict a scene region is more likely to be fixated if it is both meaningful and visually salient, while DeepGaze II prediction is associated with increasingly discounting low-level saliency as a scene region becomes more meaningful.

Finally, center proximity also played a significant role in each deep saliency model both as a fixed effect and as an interaction term. The effect of center proximity was larger in MSI-Net ($\beta ={0.38}$, CI [0.38, 0.39], $p<.001$) and SAM-ResNet ($\beta ={0.31}$, CI [0.31, 0.32], $p<.001$) compared to DeepGaze II ($\beta ={-0.01}$, CI $[-0.015, -0.010]$, $p<.001$). The interactions between center proximity and the mid-level maps (junction and symmetry maps) were very small (see Tables 2, 3, 4); however, the center proximity interactions with low-level and high-level features showed distinct patterns among the models.

**Table 2:** MSI-Net LME Results. Beta estimates ($\beta$), 95% confidence intervals (CI), standard errors (SE), *t*-statistic, and *p* values (*p*) for each fixed effect and standard deviations (*SD*) for the random effects of subject and scene.

Predictors	Fixed effects					Random effects, *SD*	
	$\beta$	95% CI	*SE*	*t*-statistic	*p*	Subject	Scene
Intercept	0.03	[− 0.04 0.10]	0.04	0.75	0.45	0.04	0.37
Meaning	0.33	[0.33 0.34]	0.002	153.13	< 0.001***	–	–
Symmetry	0.002	[− 0.003 0.008]	0.003	0.89	0.37	–	–
Junctions	0.006	[0.003 0.008]	0.001	4.27	< 0.001***	–	–
IttiKoch	0.308	[0.305 0.312]	0.002	166.54	< 0.001***	–	–
Center proximity	0.383	[0.380 0.386]	0.002	241.59	< 0.001***	–	–
Meaning: center proximity	0.092	[0.088 0.095]	0.002	52.82	< 0.001***	–	–
Symmetry: center proximity	− 0.004	[− 0.006 − 0.001]	0.001	− 2.81	< 0.01**	–	–
Junction: center proximity	0.012	[0.010 0.014]	0.001	10.28	< 0.001***	–	–
IttiKoch: center proximity	− 0.034	[− 0.036 − 0.030]	0.002	− 21.64	< 0.001***	–	–
Meaning: IttiKoch	0.044	[0.041 0.047]	0.002	25.86	< 0.001***	–	–

**Table 3:** DeepGaze II LME results.

Predictors	Fixed effects					Random effects, *SD*	
	$\beta$	95% CI	*SE*	*t*-statistic	*p*	Subject	Scene
Intercept	0.27	[0.20 0.34]	0.03	8.00	0.13	0.04	0.33
Meaning	0.448	[0.445 0.451]	0.002	288.48	< 0.001***	–	–
Symmetry	0.06	[0.05 0.07]	0.002	33.57	< 0.01**	–	–
Junctions	0.025	[0.023 0.026]	0.001	26.83	< 0.001***	–	–
IttiKoch	0.253	[0.250 0.255]	0.001	193.45	< 0.001***	–	–
Center proximity	− 0.012	[− 0.015 − 0.010]	0.001	− 11.16	< 0.001***	–	–
Meaning: center proximity	0.027	[0.025 0.030]	0.001	22.16	< 0.001***	–	–
Symmetry: center proximity	0.005	[0.003 0.007]	0.001	5.13	< 0.001***	–	–
Junction: center proximity	0.007	[0.005 0.008]	0.001	8.02	< 0.001***	–	–
IttiKoch: center proximity	− 0.034	[− 0.036 − 0.031]	0.001	-30.99	< 0.001***	–	–
Meaning: IttiKoch	− 0.078	[− 0.081 − 0.076]	0.001	-65.19	< 0.001***	–	–

Beta estimates ($\beta$), 95% confidence intervals (CI), standard errors (SE), *t* values, and *p* values (*p*) for each fixed effect and standard deviations (*SD*) for the random effects of subject and scene.

**Table 4:** SAM-ResNet LME results.

Predictors	Fixed effects					Random effects, *SD*	
	$\beta$	95% CI	*SE*	*t*-statistic	*p*	Subject	Scene
Intercept	− 0.06	[− 0.15 0.02]	0.04	− 1.53	0.13	0.05	0.42
Meaning	0.28	[0.27 0.29]	0.003	93.18	< 0.001***	–	–
Symmetry	− 0.01	[− 0.02 0.00]	0.004	− 3.14	< 0.01**	–	–
Junctions	0.016	[0.012 0.019]	0.002	8.75	< 0.001***	–	–
IttiKoch	0.274	[0.269 0.278]	0.003	107.84	< 0.001***	–	–
Center proximity	0.314	[0.310 0.318]	0.002	144.54	< 0.001***	–	–
Meaning: center proximity	0.133	[0.128 0.137]	0.002	55.82	< 0.001***	–	–
Symmetry: center proximity	− 0.001	[− 0.004 0.003]	0.002	− 0.37	0.71	–	–
Junction: center proximity	− 0.011	[− 0.014 0.008]	0.002	− 6.99	< 0.001***	–	–
IttiKoch: center proximity	0.035	[0.031 0.039]	0.002	16.72	< 0.001***	–	–
Meaning: IttiKoch	0.087	[0.083 0.092]	0.002	37.42	< 0.001***	–	–

Beta estimates ($\beta$), 95% confidence intervals (CI), standard errors (SE), *t* values, and *p* values (*p*) for each fixed effect and standard deviations (*SD*) for the random effects of subject and scene.

The interaction pattern between low-level saliency and center proximity was different in each decomposed deep saliency model. In MSI-Net ($\beta ={-0.03}$, CI $[-0.04, -0.03]$, $p<.001$), as low-level saliency increased the effect of center proximity decreased (Fig. 3b, bottom-right). In SAM-ResNet ($\beta ={0.04}$, CI [0.03, 0.04], $p<.001$), as low-level saliency increased the effect of center proximity increased (Fig. 5b, bottom-right). In DeepGaze II ($\beta ={-0.04}$, CI $[-0.04, -0.03]$, $p<.001$), a dissociation was observed. That is, as low-level saliency increased, greater center proximity switched from being associated with higher DeepGaze II values to lower DeepGaze II values (Fig. 5b, bottom-right). The interaction pattern between high-level semantic density and center proximity was consistent for MSI-Net and SAM-ResNet (MSI-Net $\beta ={0.09}$, CI [0.088, 0.095], $p<.001$; SAM-ResNet, $\beta ={0.13}$, CI [0.128, 0.137], $p<.001$). In both models, as meaning increased center proximity had a greater positive impact on the predicted deep saliency values (Fig. 3b, 5b, top-middle). In comparison, DeepGaze II showed a much smaller interaction between meaning and center proximity ($\beta ={0.027}$, CI [0.025, 0.030], $p<.001$). These different interaction patterns with center proximity are likely influenced by both the different model architectures and the different center biases in each deep saliency model.

## Discussion

Using deep saliency models to inform cognitive theories of attention requires more than state-of-the-art prediction, it requires an understanding of how that prediction is achieved. Here, we first replicated and extended to active viewing tasks that three prominent deep saliency models (i.e., MSI-Net, DeepGaze II, and SAM-ResNet) predicted where people looked in real-world scenes. Then, we decomposed the degree to which low-, mid-, and high-level scene information were associated with fixated deep saliency values. We found that MSI-Net, DeepGaze II, and SAM-ResNet are primarily learning features associated with high-level meaning and low-level saliency, and exhibited qualitatively different interaction patterns.

The present work extends our understanding of the relationship between deep saliency models, attention, and scene features in a number of important ways. First, we demonstrate how a mixed effects modeling approach can be used to directly model the association between deep saliency output and human eye behavior across low-, mid-, and high-level feature spaces. This approach is both general and flexible. That is, the mixed effects approach can be applied to any deep saliency model that produces a saliency map, any type of feature map of theoretical interest, and eye movement data from any scene viewing task. Using this approach, we show that while MSI-Net, DeepGaze II, and SAM-ResNet each predict scene attention well, they do so in qualitatively different ways. From a cognitive science perspective, this is of theoretical importance because we want to know if deep saliency models are doing what humans are doing when processing scenes. Without this information, we have no way to determine whether the features prioritized by deep saliency models to predict scene attention are similar to each other, or more importantly, if they are similar to how humans prioritize features to guide attention in scenes.

The strong association between all three deep saliency models we tested and high-level meaning suggests these deep saliency models are learning image features that are associated with scene meaning. While MSI-Net, DeepGaze II, and SAM-ResNet each have a unique architecture, training regimen, and loss function, all the models are trained on human scene fixation data. Given previous research indicating that local semantic density is one of the strongest predictors of where observers fixate in scenes^46^, it follows that deep saliency models would benefit from learning features associated with semantic density. Therefore, the use of scene fixation data to train deep saliency models may be the common factor that drives each deep saliency model to learn which pre-trained object recognition features are most associated with scene meaning. It is important to note that this does not necessarily mean that deep saliency models and human ratings of meaning are equivalent^47^. For example, recent neurocognitive work shows human-generated meaning maps produce stronger activation in cortical areas along the ventral visual stream than DeepGaze II^48^. The differences between meaning maps and deep saliency maps are most likely driven by the inherent differences between deep saliency models and human raters. Specifically, deep saliency models have a much simpler neural architecture compared to human raters, and while deep saliency models have a constrained feature set of the visual features stored in VGG-16/VGG-19, human raters likely draw on a much broader set of features including object semantics^30^.

The consistent strong association between deep saliency models and low-level image saliency is also an interesting finding. The deep saliency models each have access to low-level features in the pre-trained VGG-16 and VGG-19 weights of the models. That is, early layers of VGG-16 and VGG-19 both exhibit frequency, orientation, and color selective kernels similar to properties observed in early visual cortex^7,49,50^. Therefore, it is likely that the association we observed between low-level image saliency and each model was driven by the low-level features in the early layers of VGG-16/VGG-19 and the human fixation data during training. Interestingly, while high-level features often override low-level saliency in human observers^25–28,31^, it may be that the deep saliency models are learning when low-level features and high-level features are most relevant for predicting where people look in scenes. For example, in all three models we observed a significant interaction between low-level saliency and high-level meaning. And at least in DeepGaze II, the pattern of the interaction seemed consistent with the idea that high-level features can override low-level saliency in scenes. That is, we observed that as a fixated region’s meaning value increased, the predicted DeepGaze II values were increasingly unaffected by low-level saliency. Granted, we observed the opposite interaction pattern in MSI-Net and SAM-ResNet, so more work will be needed to understand why different deep saliency models show different interaction patterns between low- and high-level scene information. Nonetheless, these results suggest that deep saliency models are learning something about how best to prioritize low- and high-level features, although they seem to be learning different mappings in different deep saliency models.

The mid-level associations with the deep saliency models were relatively weak compared to high-level meaning and low-level saliency. This suggests that local symmetry and junction density, while significant, may only play a supporting role in attentional guidance in scenes. That is, mid-level features help to combine low-level features into higher-level representations^23^, but it is these higher-level representations that are used to determine attentional priority. Our mid-level findings using local contour symmetry and junction density complement previous work^9^ by examining how these mid-level features are directly associated with fixated deep saliency model values. Finally, it is worth noting that while our current results suggest local symmetry and junction density play marginal roles in MSI-Net, DeepGaze II, and SAM-ResNet, it may simply be that these deep saliency models are using a different kind of mid-level feature representation.

The current work has a number of limitations that would be useful to address in future work to expand our understanding of how deep saliency models predict scene attention. One limitation of the current work is that we used active viewing tasks that do not involve a specific target object (i.e., scene memorization and aesthetic judgment). The results will likely be different in a task that involves a search for a specific visual or semantic target (e.g., visual search for a a dresser in a bedroom scene). Another limitation is that the current scenes were typical indoor and outdoor scenes, without semantically inconsistent objects. So it will be important in future work to examine whether similar patterns of association hold for scenes that contain object-scene inconsistency^51–54^. Finally, we only looked at two possible mid-level features, so it would be useful in future work to test other candidate mid-level features. For example, one could investigate the intermediate layers of VGG-16/VGG-19, or other proposed mid-level feature representations such as texforms^23^. Fortunately, the general approach introduced here is flexible and can easily be applied to examine other candidate mid-level feature representations.

While deep learning models provide state-of-the-art scene fixation prediction, insights they might provide for cognitive theories of attention have been limited. In order for deep saliency models to inform cognitive theories of gaze guidance in scenes, we must find ways to understand the feature mapping these models are learning from the human data. Here, we have shown how a mixed effects modeling approach can be used to decompose the performance of deep saliency models by using maps that reflect a wide range of processing levels ranging from pre-semantic, low-level image saliency to high-level meaning. We found that all three deep saliency models were most strongly associated with high-level meaning and low-level saliency, but exhibited qualitatively different feature weightings and interaction patterns. These results highlight the importance of moving beyond simply benchmarking deep saliency models and toward understanding how deep saliency models generate their predictions in an effort to guide cognitive theory.

## Methods

### Participants

University of California, Davis undergraduate students with normal or corrected-to-normal vision participated in the eye tracking (*N* = 114) and meaning rating (*N* = 408) studies in exchange for course credit. All participants were naive concerning the purposes of the experiment and provided verbal or written informed consent as approved by the University of California, Davis Institutional Review Board.

### Stimuli

Participants in the eye tracking study viewed 100 real-world scene images. The 100 scenes were chosen to represent 100 unique scene categories (e.g., kitchen, park), where half of the images were indoor scenes and half were outdoor. Each participant in the meaning rating study viewed and rated 300 isolated, random small regions taken from the set of 100 scenes.

### Apparatus

Eye movements were recorded using an EyeLink 1000+ tower-mount eye tracker (spatial resolution 0.01$^{\circ }$) sampling at 1000 Hz^55^. Participants sat 85 cm away from a 21” monitor and viewed scenes that subtended approximately $27^{\circ } \times$ $20^{\circ }$ of visual angle. Head movements were minimized using a chin and forehead rest. Although viewing was binocular, eye movements were recorded from the right eye. The display presentation was controlled with SR Research Experiment Builder software^56^.

### Eye tracking calibration and data quality

A 9-point calibration procedure was performed at the start of each session to map eye position to screen coordinates. Successful calibration required an average error of less than $0.49^{\circ }$ and a maximum error of less than $0.99^{\circ }$. Fixations and saccades were segmented with EyeLink’s standard algorithm using velocity and acceleration thresholds (30/*s* and 9500$^{\circ }$/$s^{2}$). A drift correction was performed before each trial and recalibrations were performed as needed. The recorded data were examined for data artifacts from excessive blinking or calibration loss based on mean percent signal across trials^57^. Fourteen subjects with less than 75% signal were removed, leaving 100 subjects that were tracked well (signal mean = 92.1%, SD = 5.31%).

### Eye tracking tasks and procedure

Each participant (*N* = 100) viewed 100 scenes for 12 s each while we recorded their eye movements. Each trial began with fixation on a cross at the center of the display for 300 ms. For half the scenes, participants were instructed to memorize each scene in preparation for a later memory test. For the other half of the scenes, participants were instructed to indicate how much they liked each scene on a 1–3 scale using a keyboard press following the 12 second scene presentation. The scene set and presentation order of the two tasks were counterbalanced across subjects. This procedure produced a large eye movement dataset that contained 334,725 fixations, with an average of 3347 fixations per subject.

### Deep saliency models

We compared 3 of the best performing deep saliency models on the MIT saliency benchmark^1^: the multi-scale information network (MSI-Net)^36^, DeepGaze II^37^, and the saliency attentive model (SAM-ResNet)^38^. Each deep saliency model takes an image as input and produces a predicted saliency map as output. All of the deep saliency models were trained on human data in the form of fixation and/or mouse-contingent density maps that reflect where humans focus their attention in scenes. The model weights are fixed following training, and then the models are evaluated on new scenes and fixation data. MSI-Net, DeepGaze II, and SAM-ResNet each have distinct network architectures, training regimens, center bias priors, and loss functions which are worth considering.

#### MSI-Net

MSI-Net consists of three main components, a feature network, a spatial pooling network, and a readout network^36^. MSI-Net uses the pre-trained weights from the VGG-16 network^7^ without the feature downsampling in the last two max-pooling layers^36^. The VGG-16 network is a deep convolutional network with 16 layers and was trained on both the *ImageNet* object classification^58^ and the *Places2* scene classification datasets^59^. The activations from the VGG-16 network then feed into a spatial pooling module called the *Atrous Spatial Pyramid Pooling* (ASPP) module^60^. The ASPP module of MSI-Net has several convolutional layers which combine feature information at multiple spatial scales including a global scale to capture global scene context which has been shown to be helpful in predicting where people look in scenes^61^. Finally, the readout network contains 6 layers that include convolutional and upsampling layers and a blur. The ASPP and readout network were trained on the SALICON dataset^8^. MSI-Net prediction is optimized using the Kullback–Leibler divergence which measures the distance between the target and model estimated distributions. The MSI-Net predicted saliency maps reflect the predicted probability distribution of fixations for each scene image (Fig. 1b).

#### DeepGaze II

DeepGaze II also consists of three main components, a feature network, a readout network, and an explicit (i.e., non-learned) center bias^37^. The feature network consists of the pre-trained weights from the VGG-19 network^7^ without the fully connected layers. The VGG-19 network is a deep convolutional network with 19 layers that is trained on more than a million images to recognize 1000 different object categories from the *ImageNet* database^58^. In DeepGaze II, the VGG-19 feature network is fixed and the readout network is the only portion of the model that is trained to perform saliency prediction. The readout network consists of 4 layers that are trained on the SALICON^8^ and MIT1003^62^ datasets to predict human saliency data using the pre-trained VGG-19 features^8^. The DeepGaze II model maximizes log-likelihood and expresses saliency as probability density with blur. Finally, DeepGaze II applies a center bias to capture the tendency for observers to look more centrally in scenes^39,40^. The DeepGaze II maps reflect the predicted probability distribution of fixations for each scene image (Fig. 1c).

#### SAM-ResNet

SAM-ResNet is composed of a dilated feature network, an attentive convolutional network, and a learned set of Gaussian priors for center bias^38^. SAM-ResNet modifies the ResNet-50 network^63^ using a dilation technique to reduce the amount of input image rescaling that is detrimental to saliency prediction^64^. The ResNet-50 network is a deep convolutional network with 50 layers that is trained on the *ImageNet* object classification dataset^58^. The dilated version of the ResNet-50 feature network provides the features that then feed into the attentive convolutional network. The attentive convolutional network is a recurrent long short-term memory network (LSTM^65^) that is used to refine the most salient regions of the input regions over multiple sequential iterations. It is worth noting that this recurrent model module is fundamentally different compared to the pure feedforward MSI-Net and DeepGaze II model architectures^38^. Finally, SAM-ResNet learns a set of Guassian priors to account for observer center bias^39,40^. SAM-ResNet is trained on the SALICON dataset^8^ and uses a linear combination of multiple saliency benchmark metrics (i.e., normalized scanpath saliency, linear correlation, and Kullback-Leibler divergence^1^) as its loss function during training. The SAM-ResNet predicted saliency maps reflect the predicted probability distribution of fixations for each scene image (Fig. 1d).

### Feature maps

#### Low-level features: image saliency map

Low-level scene features were represented using the Itti and Koch model with blur with default settings^3,42,66^. Similar to other image-based saliency models, the Itti and Koch model is derived from contrasts in low-level image features including color, intensity, and edge orientation at multiple spatial scales. An image saliency map was generated for each scene stimulus and reflects the predicted fixation density for each scene based on low-level, pre-semantic image features.

**Figure 6:**
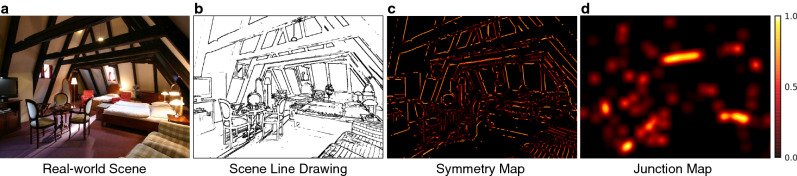
Scene, line drawing, and its corresponding symmetry and junction maps. Each scene (**a**) was first converted to a line drawing (**b**). Then, from the line drawing, local symmetry (**c**) and junction density were computed (**d**). The symmetry and junction maps served as mid-level feature maps in our analyses.

#### Mid-level features: symmetry and junction maps

Mid-level scene features were represented by two different types of maps: symmetry maps and junction maps. The symmetry and junction maps were both computed from a line drawing of each scene (Fig. 6a). The line drawings (Fig. 6b) were extracted using an automated line drawing extraction algorithm (logical/linear operators^43,67^). Then, using the contours from each scene line drawing, the symmetry (Fig. 6c) and junction (Fig. 6d) maps were computed. Each scene symmetry map reflects the degree of local ribbon symmetry of contours in the scene line drawing^43,44^. Ribbon symmetry measures the degree to which pairs of scene contours exhibit constant separation (i.e., local parallelism) along their medial axis^43,44^. Each scene junction map shows the density of points where at least two separate scene contours intersect each other^68^.

#### High-level features: meaning map

Meaning maps were generated as a representation of the spatial distribution of high-level, semantic density (^26,27^; see https://osf.io/654uh/ for code and task instructions). Meaning maps were created for each scene by cutting the scene into a dense array of overlapping circular patches at a fine spatial scale (300 patches with a diameter of 87 pixels) and coarse spatial scale (108 patches with a diameter of 207 pixels). Each rater (*N* = 408) then provided ratings of 300 random scene patches based on how informative or recognizable they thought they were on a 6-point Likert scale^24,26^. Patches were presented in random order and without scene context, so ratings were based on context-independent judgments. Each patch was rated by three unique raters. A meaning map (Fig. 1f) was generated for each scene by averaging the rating data at each spatial scale separately, then averaging the spatial scale maps together, and then smoothing the grand average rating map with a Gaussian filter (i.e., Matlab ’imgaussfilt’ with $\sigma =10$, *FWHM* = 23 px).

#### Center proximity map

In addition to the low-, mid-, and high-level feature maps, we also generated a center proximity map that served as a global representation of how far each location in the scene was from the scene center. Specifically, the center proximity map measured the inverted Euclidean distance from the center pixel of the scene to all other pixels in the scene image (Fig. 1e). The center proximity map^30^ was used to explicitly control for the general bias for observers to look more centrally than peripherally in scenes, independent of the underlying scene content^39,40^.

### Statistical models

### Fixated and non-fixated scene locations

We modeled the association between the eye movement data and each deep saliency model by comparing where each subject looked in each scene to where they did not look^30,41^. Specifically, for each region a subject fixated, we computed the mean value for each deep saliency model (Fig. 1b–d) and the center proximity map (Fig. 1e) by taking the average over a $3^{\circ }$ window around each fixation (Fig. 1a, neon green locations). To represent the model and center proximity values that were not associated with overt attention, for each individual subject, we randomly sampled an equal number of scene locations where each subject did not look in each scene they viewed (Fig. 1a, cyan locations). The only constraint for the random sampling of the non-fixated scene regions was that the non-fixated $3^{\circ }$ windows could not overlap with any of the fixated $3^{\circ }$ windows. This procedure was performed separately for each individual scene viewed by each individual subject.

### General linear mixed effects models: how well do deep saliency models predict scene attention?

We applied a general linear mixed effects (GLME) logit model to examine how well each deep saliency model accounted for the eye movement data using the *lme4* package^69^ in R^70^. We used a mixed effects modeling approach because it does not require aggregating the eye movement data at the subject or scene-level like ANOVA or map-level correlations. Instead, both subject and scene could be explicitly modeled as random effects. The GLME approach allowed us to control for center bias by including the center proximity (Fig. 1e) of each fixated and non-fixated region as both a fixed effect and as an interaction term with the deep saliency model values. Specifically, whether a region was fixated (1) or not fixated (0) was predicted as a function of the fixed effects of each respective deep saliency map value (i.e., MSI-Net, DeepGaze II, or SAM-ResNet), center proximity value, and the deep saliency model by center proximity interaction. Subject and scene were treated as random intercepts. Since we are interested in how well each deep saliency model performs generally, regardless of task, the memorization and aesthetic judgment data were combined in all models. To compare the performance of the three different deep saliency models, a GLME model was fit separately for each deep saliency model (see Fig. 2 and Table 1).

### Linear mixed effects models: how do deep saliency models weight low-, mid-, and high-level features?

We quantified the associations between low-, mid-, and high-level features and each deep saliency mode by fitting a linear mixed effects (LME) model to each deep saliency model using the *lme4* package^69^ in R^70^. In each LME model, the fixated deep saliency model values (i.e., MSI-Net, DeepGaze II, or SAM-ResNet) were modeled as a function of the fixed effects of center proximity (bias), Itti and Koch image saliency (low-level), symmetry and junction (mid-level), and meaning (high-level). Given the known strong effect of center bias^39,40^ we included center proximity as an interaction term with all other feature maps. Finally, since high-level and low-level features are known to be associated with each other^26,32^, we included a low-level by high-level feature interaction term (i.e., Itti & Koch X Meaning) in each deep saliency LME model. Conceptually, these LME models for each deep saliency model (MSI-Net, Fig. 3; DeepGaze II, Fig. 4; SAM-ResNet, Fig. 5) estimate the degree to which the various feature maps (i.e., low-, mid-, and high-level) are related to the respective deep saliency model output.
